# The Efficacy of Yttrium-90 Radiosynovectomy in Patients with Camptodactyly-Arthropathy-Coxa Vara-Pericarditis Syndrome

**DOI:** 10.4274/mirt.29484

**Published:** 2017-02-01

**Authors:** Sulaiman Mohammed Al-Mayouf, Nora Almutairi, Khalid Alismail

**Affiliations:** 1 King Faisal Specialist Hospital and Research Center, Clinic of Pediatric Rheumatology, Riyadh, Saudi Arabia; 2 King Faisal Specialist Hospital and Research Center, Clinic of Musculoskeletal Radiology, Riyadh, Saudi Arabia

**Keywords:** Camptodactyly-arthropathy-coxa-vara-pericarditis, Radiosynovectomy, yttrium-90

## Abstract

**Objective:**

Camptodactyly-arthropathy-coxa-vara-pericarditis (CACP) syndrome is an autosomal recessive disorder caused by mutations in *PRG4* gene that encodes for proteoglycan 4, the main lubricant for joints and tendon surfaces. It is a non-inflammatory arthropathy, characterized by joint effusions and synovial hypertrophy. So far, there is no effective treatment for this disorder. To evaluate the effectiveness of yttrium-90 radiosynovectomy in arthropathy of patients with CACP syndrome.

**Methods:**

Consecutive patients with CACP syndrome were prospectively evaluated at the enrollment and 3 months after the right knee injection with yttrium-90. The outcome variables were patient/parent and physician’s global assessment measured by a 3-point scale, right knee swelling and range of motion on a 3-point scale, in addition to magnetic resonance imaging (MRI) assessment of the right knee for bone, cartilage, fluid, synovial hypertrophy and soft tissue changes.

**Results:**

Six (three boys, three girls) patients with a mean age of 12 years and mean follow-up duration of 8.5 years completed a single right knee intra-articular yttrium-90 injection with 5 mCi. The procedure was well tolerated without adverse events apart from mild and transient joint pain in two patients. There was a minimal radioisotope leakage to soft tissue in two patients. During the 3-month follow-up interval, there was no improvement in the outcome variables. Patients and parents did not notice favorable therapeutic effects and global physician assessment was unsatisfactory. There was no difference in knee joint swelling or range of motion. Furthermore, MRI findings were unchanged. However, there was a minimal increase in synovial fluid post injection.

**Conclusion:**

Yttrium-90 radiosynovectomy seems to be a safe and well tolerated procedure, however, it did not show a beneficial therapeutic effect in arthropathy of CACP syndrome with the given dosage and interval. Studies including a larger number of patients and probably repeated injections are needed to derive satisfactory results about the effectiveness of yttrium-90 in CACP syndrome patients.

## Abstract (tr)

**Amaç:**

Kamptodaktili-artropati-koksa vara-perikardit (CACP) sendromu eklem ve tendon yüzeylerinin ana lubrikanı olan proteoglikan 4’ü kodlayan *PRG4* genindeki mutasyonlara bağlı bir otozomal resesif hastalıktır. Eklemde efüzyon ve sinovyal hipertrofi ile karakterize non-enflamatuvar bir artropatidir. Henüz bu hastalığın etkin bir tedavisi yoktur. Amacımız CACP sendromlu hastaların artropatisinde yttrium-90 radyosinovektominin etkinliğini araştırmaktır.

**Yöntem:**

CACP sendromu tanısı alan ardışık hastalar tanı anında ve sağ dize yttrium-90 enjeksiyonu uygulanmasından 3 ay sonra prospektif olarak değerlendirildiler. Sonuç değişkenleri 3-puanlı skala üzerinden hasta/ebeveyn ve doktor genel değerlendirmesi, 3-puanlı skala üzerinden sağ diz ödemi ve hareket aralığı ve kemik, kıkırdak, sıvı, sinovyal hipertrofi ve yumuşak doku değişiklikleri açısından sağ diz manyetik rezonans görüntülemesi (MRG) idi.

**Bulgular:**

Ortalama yaşı 12, ortalama takip süreleri 8,5 yıl olan altı hastaya (üç erkek, üç kız) tek sefer sağ dize 5 mCi intra-artiküler yttrium-90 enjeksiyonu uygulandı. İşlem iki hastada hafif ve geçici eklem ağrısı dışında herhangi bir komplikasyon olmadan iyi tolere edildi. İki hastada yumuşak dokuya minimal radyoizotop kaçağı oldu. Üç aylık takip süresince sonuç değerlerinde iyileşme olmadı. Hastalar ve ebeveynleri olumlu terapötik etki fark etmediler ve genel doktor değerlendirmesi tatmin edici değildi. Diz eklemi ödemi ve hareket aralığında değişiklik yoktu. MRG bulguları değişmedi, ancak enjeksiyon sonrası sinovyal sıvıda minimal artış saptandı.

**Sonuç:**

Yttrium-90 radyosinovektomi güvenli ve iyi tolere edilen bir işlem olarak görülmektedir, ancak verilen doz ve sürede CACP sendromlu hastaların artropatisinde yararlı bir terapötik etki göstermemiştir. Daha fazla hasta içeren ve muhtemelen tekrarlayan enjeksiyonların uygulandığı çalışmalar yttrium-90’ın CACP sendromlu hastalardaki etkinliği ile ilgili gerekli sonuçları sağlayabilir.

## INTRODUCTION

Camptodactyly-arthropathy-coxa vara-pericarditis (CACP) syndrome is one of the autosomal recessive familial arthropathies (1,2,3,4). Typically, patients with CACP syndrome present with articular features mimicking most common rheumatic disorders, it is not unusual to mistake these disorders as juvenile idiopathic arthritis (JIA) (5). The locus of CACP syndrome was allocated to a 1.9-cm interval on human chromosome 1q25-31 by homozygosity mapping, and proteoglycan 4 (*PRG4*) was identified as the responsible gene (4,6). Furthermore, mutations in the gene encoding the secretion of *PRG4* lead to synovial hyperplasia and loss of its lubricating function, which is the principal pathological feature of this syndrome (7,8). Currently, there are 15 reported *PRG4* mutations (9,10,11).

CACP syndrome is a rare entity, and its worldwide frequency is yet unknown. Although, it has been described in different ethnicities, the diagnosis of CACP syndrome in Saudi families is relatively frequent (9,12,13,14). Unfortunately, there is no available effective treatment yet.

Medical synovectomy (radiosynovectomy) using radioactive isotope is considered as an alternative therapeutic option for different chronic inflammatory arthritis pathologies such rheumatoid arthritis, and osteoarthritis. Radiosynovectomy is also used as an adjuvant therapy in patients with pigmented villonodular synovitis and hemophilic arthropathy (15,16,17,18,19,20). It seems that radiosynovectomy was safe and highly beneficial to children with hemophilic arthropathy. To the best of our knowledge, radiosynovectomy has not been used in CACP syndrome patients. We conducted this study to assess the effectiveness of radiosynovectomy in the treatment of knee arthropathy, using yttrium-90 in patients with CACP syndrome.

## MATERIALS AND METHODS

**Figure 1:**
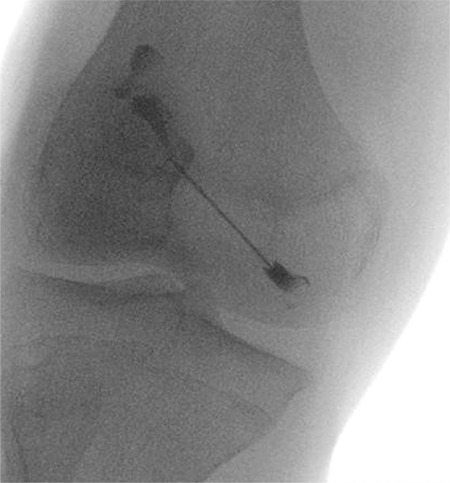
Anterior-posterior oblique projection shows the introduced needle from the medial side with the iodinated contrast

Consecutive patients with CACP syndrome seen in pediatric Rheumatology clinic at King Faisal Specialist Hospital and Research Center, (KFSH-RC), Riyadh, between May 2015 and March 2016 were included. All involved patients had thorough history and physical examination and the basic blood tests, including complete blood counts, renal and hepatic profile, as well as magnetic resonance imaging (MRI) of both knees at enrollment and 3 months after the therapeutic intervention. The expert musculoskeletal radiologist performed yttrium-90 intra-articular injection of the right knee under fluoroscopy to ensure that the needle was correctly positioned under aseptic circumstances, and the dosage of yttrium-90 was 5 mCi (Figure 1). Following injection of yttrium-90, frontal and lateral scintigraphy was performed to check the distribution of the radioactive material in the joint. Long-acting glucocorticoids (kenalog 1 mg/ kg) was injected to reduce the risk of acute synovitis. Furthermore, following the procedure, the joint was immobilized by an elastic bandage and the patient was confined to bed for 3 days.

The outcome variables were the patient/parent and the physician’s global assessment, range of motion, and swelling as well as MRI findings of the right knee. The parents/patients and the physician completed the global assessment as measured by a 3-point scale (improved, no change, worse). Right knee swelling and range of motion, which was documented by a physical therapist, were assessed on a 3-point scale (improved, no change, worse) in addition to the right knee MRI findings including the bone, cartilage, fluid, synovial hypertrophy and soft tissue changes. Similar assessments were completed 3 months after yttrium-90 intra-articular injection.

All collected data were saved and the confidentiality of the patients protected. Personal identifying data were not collected for this research project. The Research Advisory Council and the Ethical Committee of the KFSH-RC approved the study (#2020023). Informed consent was obtained from each participant.

The results were expressed as mean + standard deviation for continuous variables and percentages for categorical variables. A p value <0.05 was considered as significant. The variables were compared using 2-sample t-tests, chi-square tests and Fisher’s exact tests.

## RESULTS

Six (three boys, three girls) CACP syndrome patients with a mean age of 12 (7-20) years and a mean follow-up duration of 8.5 (3-11) years were included. The clinical and genetic findings of all patients were previously described (10,11,12). At the time of enrollment, all patients had bilateral flexion contracture and limited extension of knee joints. Additionally, they had significant swelling of the knee joint with large effusion and thickened rubbery synovium. There was no associated pain or tenderness on joint motion.

**Figure 2:**
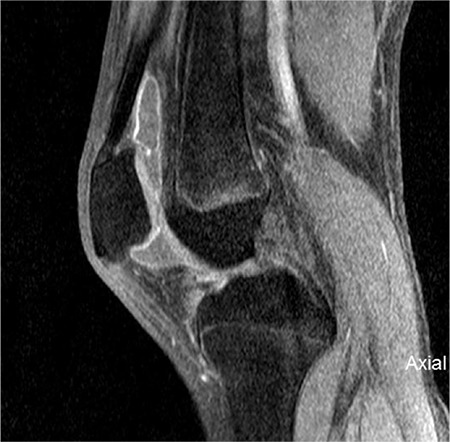
Sagittal T1 fat-saturated weighted magnetic resonance imaging images post contrast injection shows synovial hypertrophy and mild synovial enhancement. Note the amount of synovial fluid

**Figure 3:**
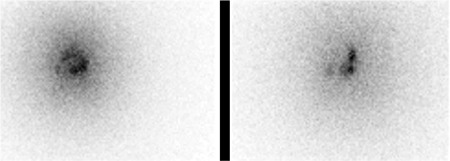
Gamma camera images showing the distribution of yttrium-90 within the joint

All patients had a normal complete blood count, renal and hepatic profile and acute phase reactants. MRI prior to yttrium-90 injection showed moderate to severe knee joint effusion with thickened enhanced synovium (Figure 2). The procedure was well tolerated without significant adverse events, gamma camera scans post yttrium-90 injection showed intra-articular homogenous distribution of the radioisotope. However, two patients had minimal leakage to soft tissue (Figure 3).

**Figure 4:**
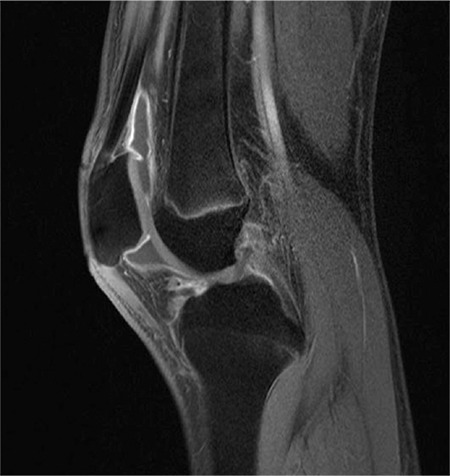
Sagittal T1 weighted fat-saturated magnetic resonance imaging images, post yttrium-90 therapy 2.8 mCi (millicurie)

Outcome variables did not change significantly 3 months after post yttrium-90 injection. The patients and parents did not notice favorable therapeutic effects, there was no significant improvement in the global assessment of the parent/patients. Furthermore, the global physician assessment was unsatisfactory. Additionally, the range of motion of the right knee was almost the same and there was no difference in knee joint swelling. Moreover, MRI findings remained unchanged. However, there was a minimal increase in synovial fluid post injection (Figure 4).

## DISCUSSION

CACP syndrome is a rare autosomal recessive non-inflammatory arthropathy with typical musculoskeletal manifestations, particularly coxa vara and multiple joint contractures.

Affected individuals usually suffer from limited range of motions, mainly the large joints, which might interfere with daily activities. Synovial hyperplasia and loss of the lubricating function is the pathological feature of this syndrome.

Taking the pathophysiology of the disease into consideration, it is predictable that CACP syndrome patients did not respond to anti-inflammatory medications. Actually, some patients with CACP syndrome were misdiagnosed as JIA and were treated with methotrexate and biologic therapy, but without beneficial therapeutic effects.

Despite the availability of effective medical treatment, including systemic anti-rheumatic and local articular treatment of inflammatory arthritis, other therapeutic interventions have been explored particularly in refractory cases. Historically, surgical synovectomy was one of the therapeutic options. However, such an intervention may induce further articular damage and complications. Radiosynovectomy, which is a less invasive procedure is considered as an alternative therapeutic option for chronic inflammatory arthritis (15,16,17,18,19,20). Radiopharmaceutical excretion is not a concern since the application is local.

We have the privilege at KFSH-RC to follow the largest cohort of children with CACP syndrome (21). Typically, the synovial histopathology showed proliferating epithelium with moderate fibro-collagenous densities and multinucleated giant cells. The long-standing disease is mostly complicated by irreversible articular changes in the form of multiple joints stiffness and contractures, and bone dysplasia such as flattening of the femoral heads, widening of the femoral necks with osteophyte formations and secondary degenerative changes (22). We were hoping that the radioactivity concentrates in the synovium would induce a necrosis of the proliferating synoviocytes. Unfortunately, yttrium-90 radiosynovectomy did not show a beneficial therapeutic effect in our patients. Nonetheless, the procedure was safe and well tolerated. Previous studies of radiosynovectomy in children with hemophilic arthropathy showed encouraging results. However, it is worth mentioning that most patients with hemophilic arthropathy underwent more than one yttrium-90 intra-articular injections (23,24). It is advised to perform radiosynovectomy for hemophilic arthropathy without delay and before the synovitis becomes severe and chronic, otherwise the response to yttrium-90 injections would decrease. Interestingly, other studies revealed that the overall success rate for radiosynovectomy was related to the underlying disease: the treatment was more effective among patients with rheumatoid arthritis and less effective for patients with arthritis of unknown origin (25). Our results might be explained by the difference in the pathology of CACP syndrome. Furthermore, disease stage, particularly the severity of synovial hyperplasia and thickened synovium may be inversely related to the clinical response in addition to the applied dosage and interval of treatment.

## CONCLUSIONS

Yttrium-90 radiosynovectomy seems to be a safe and well tolerated procedure, however, it did not show a beneficial therapeutic effect in arthropathy of CACP syndrome with the given dosage and interval. Studies including a larger number of patients and probably repeated injections are needed to derive satisfactory results about the effectiveness of yttrium-90 in patients with CACP syndrome.
